# Protective immunity to liver-stage malaria

**DOI:** 10.1038/cti.2016.60

**Published:** 2016-10-21

**Authors:** Lauren E Holz, Daniel Fernandez-Ruiz, William R Heath

**Affiliations:** 1 Department of Microbiology and Immunology, The Peter Doherty Institute, University of Melbourne, Melbourne, Victoria, Australia; 2 The ARC Centre of Excellence in Advanced Molecular Imaging, University of Melbourne, Melbourne, Australia

## Abstract

Despite decades of research and recent clinical trials, an efficacious long-lasting preventative vaccine for malaria remains elusive. This parasite infects mammals via mosquito bites, progressing through several stages including the relatively short asymptomatic liver stage followed by the more persistent cyclic blood stage, the latter of which is responsible for all disease symptoms. As the liver acts as a bottleneck to blood-stage infection, it represents a potential site for parasite and disease control. In this review, we discuss immunity to liver-stage malaria. It is hoped that the knowledge gained from animal models of malaria immunity will translate into a more powerful and effective vaccine to reduce this global health problem.

## Introduction

Malaria is the most prevalent parasitic infection in the world and a major cause of mortality, with 214 million cases of disease and ~438 000 deaths in 2015.^1^ Malaria is endemic in equatorial regions, including parts of Asia, South and Central America, and much of Africa, with 88% of cases occurring in the latter.^1^ Disease in humans is caused by five species of the *Plasmodium* parasite, *P. falciparum, P. vivax, P. malariae, P. ovale* and *P. knowlesi,* and is transmitted by bites from the female Anopheles mosquito. *P. falciparum* and *P. vivax* are the most prevalent species to cause disease with *P. falciparum* causing the highest mortality.^1^ Current controls to limit infection include the use of insecticide-treated bed nets and anti-parasite drugs such as chloroquine and artemisinin-based combination therapies (ACTs). However, long-term use of ACTs is not cost-effective and resistance to these drugs is emerging.^2^ Thus, development of an effective malaria vaccine would be ideal.

**Figure 1:**
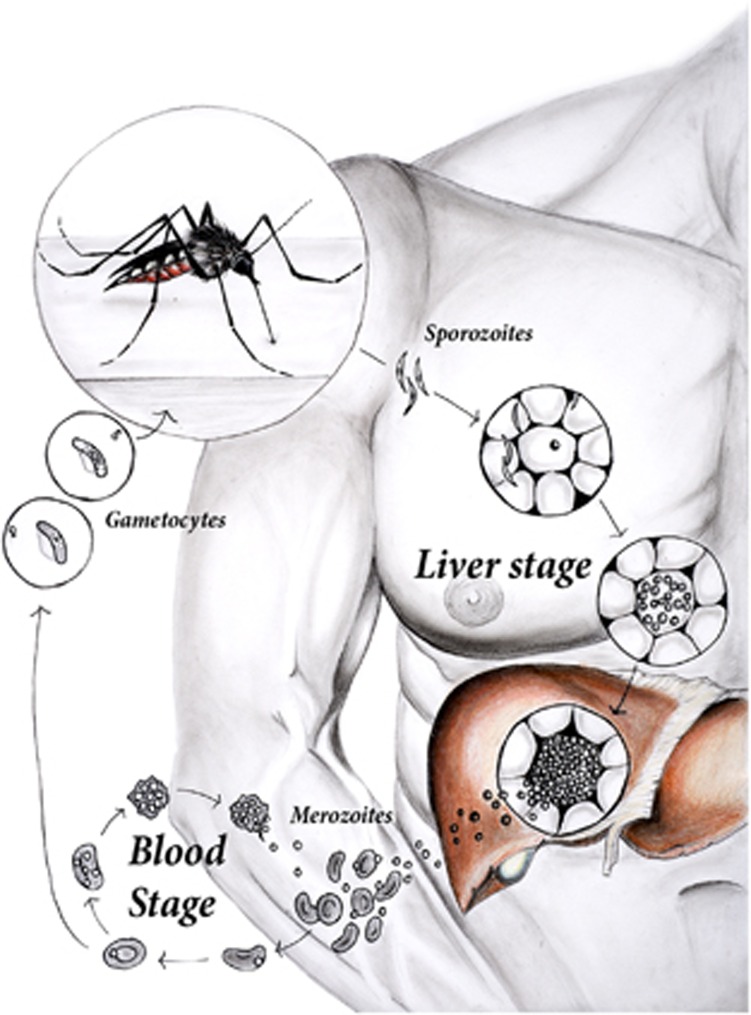
Sporozoites are introduced into the skin following a bite from an infected Anopheles mosquito and within a few hours migrate via the blood to the liver where they infect hepatocytes. During the liver phase of disease, which lasts approximately one week in humans, the sporozoites undergo asexual replication and maturation where sporozoites differentiate into schizonts. Eventually the schizont releasing thousands of merozoites into the blood. Merozoites infect red blood cells and undergo another series of asexual replication every 48 hours but this timing may vary depending on the species of *Plasmodium*. At this stage, the red blood cell bursts and the cycle begins again thus parasite numbers increase every 2 days. Other merozoites develop into immature gametocytes during the blood stage. If a mosquito bites an infected person the gametocytes can be taken up during the blood meal and mature into sporozoites in the mosquito gut. Thus the mosquito acts as a vector transmitting the disease from one human to another.

One of the most problematic hurdles to overcome in developing a malaria vaccine is the highly complex lifecycle of the parasite (Figure 1). The pre-erythrocytic phase that lasts for approximately a week in humans (with some variation between *Plasmodium* species) can be split into the early sporozoite stage, which lasts for minutes to hours, and the liver stage, which takes the bulk of this time. Sporozoites are introduced into the skin following a bite from an infected mosquito and migrate via the blood to the liver where they infect hepatocytes (reviewed in ref. 3). During the liver phase, the sporozoites undergo asexual replication and maturation where sporozoites develop into schizonts. Eventually, the schizont releases thousands of merozoites into the blood, thus initiating the blood stage of infection. Merozoites infect red blood cells (RBCs) and undergo asexual replication in humans every 24–72 h (depending on the *Plasmodium* species) producing 8–24 new merozoites.^4^ Some merozoites develop into immature gametocytes during the blood stage and, if a mosquito bites an infected person, these gametocytes can be taken up during the blood meal and later develop into sporozoites in the mosquito host (reviewed in ref. 3).

Many studies have focused on the liver stage of the disease as this point represents a bottleneck for the parasite. Relatively low numbers of parasites are found at this asymptomatic stage and pre-existing memory T-cell responses have the potential to eliminate infected hepatocytes, preventing parasite transition to the blood stage.^5^ In this review, we will briefly discuss the unique nature of the liver and how studies in animal models have identified CD8^+^ T cells as being necessary for protective immunity during liver stage infection. We will also focus on memory CD8^+^ T-cell responses, which have consistently been found to be critical for sterile immunity or complete protection. Using knowledge gained from animal models of malaria and the data generated thus far from whole-sporozoite human vaccine trials, it is hoped that an effective long-lasting malaria vaccine can be generated.

## The architecture of the liver

The basic structural unit of the liver is the hepatic lobule comprised of plates of hepatocytes (1–2 cells thick) arranged around a central vein. Hepatocytes radiate from the central vein forming a hexagon shape with the portal triads located in the corners. Portal triads consist of a branch of the hepatic artery, portal vein and a bile duct. Approximately 20% of the hepatic blood volume arrives from the hepatic artery and mixes in the liver sinusoids with the remaining 80% of blood from the portal vein.^6^ The large volume of blood traversing the liver (1.5 l of blood per minute in humans) passes through the hepatic sinusoids, which are the capillary bed of the liver. Sinusoids differ markedly in structure from the capillaries in other organs; as unlike endothelial cells in other organs, liver sinusoidal endothelial cells do not form tight junctions and have fenestrations that are grouped in clusters known as sieve plates.^7^ Furthermore, unlike other organs, liver-sinusoidal endothelial cells and hepatocytes are not separated by a basement membrane; rather, they line a gap known as the Space of Disse in which Stellate cells reside.^8^ These features make the endothelial layer in the liver more porous compared with other organs.^9^ In addition, the blood flow through the liver is reported to be slow compared with other solid organs due to the high vascularity of the liver and the large surface area of the sinusoids.^10^ Hepatic blood flow may also be influenced by the presence of hepatic macrophages, or Kupffer cells (KCs), which patrol the sinusoids and can partially occlude the lumen.^11^ Once the blood has passed through the sinusoids, it collects in the central vein and recirculates to the heart.

The distinctive architecture of the liver, with its fenestrated endothelium, allows for cognate interactions between T cells and the major organ stroma that is, hepatocytes, a process that usually does not occur in other tissues.^12, 13, 14^ Moreover, this unique structure means that it is not necessary for T cells to extravasate across the endothelial layer to eliminate infected hepatocytes.^15^ Thus, the unique nature of the liver may allow for efficient monitoring and elimination of infectious agents by CD8^+^ T cells that move through the liver sinusoids. Below, we will discuss how parasites infect the liver and how effective anti-parasite CD8^+^ T-cell responses can be generated to kill the parasite.

## Malaria liver-stage infection

Following a bite from a mosquito, it is estimated that around 100 sporozoites are introduced into the skin.^16, 17^ Approximately half of these will eventually leave the skin within 2 h^17, 18^ and travel via the blood to the liver, but the number of sporozoites that infect the liver is less clear. How sporozoites gain access to the hepatocytes is controversial, with conflicting evidence for the role of KC in this process. Heparan sulfate proteoglycans that project through fenestrations in the endothelial layer in the liver can ‘catch' sporozoites in the sinusoids, leading to direct infection of hepatocytes.^19^ Alternatively, parasites can use a gateway through KC to gain access to the parenchyma.^20^ The apical portion of the sporozoite can position itself against the KC and traverse it.^21^ Although sporozoites can be observed within vacuoles in the KC, they do not colocalise with lysosomal markers^22^ indicating that the sporozoites are not degraded. Rather, the parasites traverse KC, cross the endothelial layer, increase in speed and enter hepatocytes. The requirement for KC in parasite infection of hepatocytes has been supported by intravital studies showing sporozoites passing through KC to gain access to the hepatocytes under the endothelial layer.^23^ However, other studies using clodronate liposomes to deplete KC found greater levels of liver infection in the absence of KC, suggesting these cells are not required for hepatocyte infection.^24^ Recent data generated in support of the gateway model identified a synthetic protein P39 by phage display that blocked sporozoite interactions with KC.^25^ P39 was shown to bind CD68 on KC,^25^ suggesting it may be the putative receptor for sporozoite invasion.^21, 22^ Infection of wild type and CD68^−/−^ mice with *P. berghei* sporozoites resulted in a large reduction in parasite burden in the liver of knockout mice compared with controls supporting the view that a high proportion of sporozoites use this receptor on KC to invade the liver.^25^ Interactions between sporozoites and KC not only appear to facilitate liver invasion but may result in KC apoptosis,^26^ which may also impair potential antigen presentation by this cell type.

Parasites do not remain in the first hepatocyte they enter but pass through many, over several minutes, before settling in a final hepatocyte.^21^ Within that cell, the sporozoite establishes a parasitophorous vacuole and undergoes rounds of asexual replication. After about 2 days in mice and around a week in humans, up to 30 000 merozoites are released from each infected hepatocyte into the bloodstream,^27^ and these mature parasites are able to infect RBCs. In mice, it is estimated that only 1 in one million hepatocytes are infected whereas in humans this decreases to one in 100 million.^28^

Due to the low number of infected (and antigen presenting) hepatocytes and the short liver phase of the infection, it is vital that the immune system be well prepared to eliminate the small number of parasites that escape from the skin and migrate to the liver.

## Generating an immune response to liver-stage infection

### Innate immunity

The liver phase of the malaria lifecycle has traditionally been viewed as clinically silent due to a lack of overt symptoms. Recent data suggest that although patients might not display symptoms at this stage, a complex series of events are occurring to initiate the immune response.

Studies in mice using *P. berghei* and *P. yoelii* have revealed that parasites can trigger the type I interferon (IFN) transcription programme in the infected hepatocytes.^29^ Unlike viruses and bacteria, malaria parasites can trigger type I IFNs in the absence of Toll-like receptors (TLR3 and TLR4) and their signalling proteins (MyD88 and TRIF). Rather malaria parasites are detected within the infected hepatocytes via melanoma differentiation-associated gene 5 protein (MDA5) and signalling of this recognition occurs via the mitochondrial antiviral signalling protein (MAVS), which activates the transcription factors IRF3 and IRF7. In addition to hepatocytes, type I IFN signalling was also evident in myeloid cells and this process resulted in the recruitment of lymphoid cells to the liver. These cells were often found around infected hepatocytes but were not present in the absence of this pathway indicating that the liver phase is not inert.^29^

Natural killer (NK) and natural killer T (NKT) cells are abundant in the livers of humans and mice, and are ideally situated to interact with malaria parasites and initiate liver-stage cell-mediated immunity. NK cells increase in the liver following *P. yoelii* infection,^30^ and *in vitro* studies show that they can inhibit the development of parasites in hepatocytes but have no effect on the blood stage.^30^ NKT cells can also have a direct effect on liver-stage parasites for a brief period upon stimulation, as mice treated with α-GalCer, an NKT cell-specific ligand, had reduced parasite rRNA compared with controls.^31^ Indeed, NKT cell numbers increase in the liver after infection with high numbers of attenuated *P. yoelii* parasites and can contribute to the control of wild-type sporozoite infection 3 days later.^32^ Moreover, NKT cells may contribute to CD8^+^ T-cell accumulation in the liver after some forms of vaccination.^31, 33^ However, radiation-attenuated sporozoite (RAS)-vaccinated CD1d deficient mice, which lack NKT cells, show similar protection rates to wild-type mice after *P. berghei* sporozoite challenge,^34^ indicating NKT cells are not required for protection under physiological conditions. Furthermore, CD1d knockout mice or mice depleted of NKT cells display normal CD8^+^ T-cell responses following RAS vaccination indicating NKT cells do not provide help to CD8^+^ T cells.^35, 36^

### Priming the adaptive immune system for liver-stage immunity

Given the poor ability of the steady-state innate immune system to effectively control sporozoites, the adaptive arm of the immune system must be triggered for long-lived protection. The first signs of T-cell activation during natural infection are observed in the skin draining lymph nodes (LN) 2 days after a mosquito blood meal.^37^ Sporozoites can be detected in these LN by PCR as early as 6 h post inoculation^18^ and priming at this site enhances the intrahepatic immune response as removal of the draining LN prior to inoculation reduces the number of activated T cells in the liver by 2-fold.^37^ Antigen-specific CD8^+^ T cells tend to cluster around CD8^+^ dendritic cells (DC) but not around CD11b^+^ DC after sporozoite infection,^38^ and restricting antigen presentation to the skin draining LN reveals that T-cell activation in the LN alone is sufficient to provide protection against malaria.^39^ In these experiments, wild type and CD81^−/−^ mice (in which parasites cannot establish infection in the liver and hence there is no direct antigen presentation in the liver) were vaccinated with RAS. Following vaccination, splenocytes from these mice were transferred into naïve recipients that were subsequently challenged with sporozoites. All mice regardless of the source of donor cells were protected from challenge thus T-cell priming in the LN is sufficient for protection.^39^ In some circumstances, T-cell priming can also be observed in the spleen and liver but this aligns with situations where parasites are delivered intravenously (i.v) rather than via their natural route.^40, 41^

Extensive studies using transgenic mouse models have investigated the role of liver cells in direct antigen presentation of parasite epitopes to naïve CD8^+^ T cells as about 85% of sporozoites that leave the skin, enter the blood stream and potentially reach the liver.^42^ Responding transgenic circumsporozoite protein (CSP)-specific CD8^+^ T cells first appear in the liver ~3 days after parasite exposure,^37, 40^ suggesting these cells are primed elsewhere and recirculate to the liver. In fact, pretreating splenectomised mice with FTY720, a drug that blocks lymphocyte egress from LNs, decreases transgenic T-cell numbers in the liver by over 80%.^37^ Consistent with this data, lymphadenectomy reduced T-cell numbers in the liver by over 60%, and when both LNs and the spleen were removed the number of transgenic T cells in the liver fell by 80%,^37^ suggesting the LNs are the major site of activation. The small number of activated transgenic cells present in the liver in splenectomised and lymphadenectomised mice were most likely activated *in situ* by hepatocytes as CSP, the antigen recognised by the transgenic T cells, can be degraded within hepatocytes and presented on MHC class I molecules to CD8^+^ T cells, though it is not cross-presented by these cells.^43, 44^ Given the location of liver DC around portal tracts, it is unlikely that naïve CD8^+^ T cells would have access to this site and be primed by these cells.^45^ The role of KCs in T-cell priming is less clear as these cells are readily accessible to naïve T cells in the sinusoids, interact with the parasites prior to invasion of hepatocytes^25^ and have elevated levels of MHC I postinfection.^46^ Further investigation will be required to determine if KCs can directly activate parasite-specific CD8^+^ T cells in the liver or if they are killed after exposure to sporozoites as suggested in one study.^26^ Combined, these data indicate that skin draining LN are the major site of T-cell priming after *Plasmodium* infection, with a smaller contribution by the spleen and hepatocytes.

Following T-cell priming in skin draining LN, activated CD8^+^ T cells migrate to the liver and seek out parasites. A ‘liver homing' marker has not been conclusively identified but this process may involve CXCR6, as its ligand CXCL16 is highly expressed on liver sinusoidal endothelial cells and has been implicated in the retention of NKT cells in this organ.^47^ Transfer of CXCR6-deficient transgenic T cells from ovalbumin-specific OT-I mice prior to vaccination with RAS expressing SIINFEKL (the antigen recognised by OT-I cells) results in a 75% reduction in T cell numbers in the liver at day 25.^48^ The small number of T cells in the liver at this stage may have been retained by CXCR3 interactions as liver memory CD8^+^ T cells have been found to express high levels of CXCR3 compared with splenic populations. Bone marrow chimera studies revealed effector CD8^+^ T cells must recognise antigen on hepatocytes to eliminate the parasites. KC and DC were not required for this process.^37^ In support of this view, co-transfer of infected hepatocytes and effector CSP-specific T cells into transporter associated with antigen processing (TAP) knockout mice resulted in 100% protection,^49^ indicating that in the absence of antigen presentation by APC of the recipient mice, direct presentation by infected hepatocytes is sufficient for protection.

Combined, these data show that while bone marrow-derived cells are required for the initial priming of parasite-specific CD8^+^ T cells, effector T-cell reactivation in the liver is mediated by hepatocytes and this process is essential for the elimination of the parasites.

## Parasite-specific CD8^+^ T-cell responses in the liver

The pivotal role of CD8^+^ T cells in protective immunity was first demonstrated in the 1980s using mice vaccinated with RAS.^50^ Immunisation of this type induced sterile immunity but was lost when CD8^+^ T cells were depleted from the T-cell repertoire.^50, 51, 52^ To achieve sterile immunity, large numbers of parasite-specific CD8^+^ T cells are required and it is estimated that these numbers are 100–1000-fold higher than those that would be required for sterile immunity against viral or bacterial pathogens.^5, 53^ Why such large numbers are required is not entirely clear but a prevailing view has been that it relates to the very short duration of the liver stage, in which time every single infected hepatocyte must be eliminated or else overt blood-stage malaria will ensue.

Naive CD8^+^ T cells are not well equipped to deal with sporozoites due to their low frequency and lack of effector functions. In fact, naïve CSP-specific transgenic CD8^+^ T-cell proliferation cannot be detected until 2 days post infection^41^ and hence this population is unable to amass significant numbers to fight the parasites within the 2-day time frame required for parasites to egress from the liver, at least in mice. Moreover, naive cells are unable to generate effector functions such as IFN-γ until 24 h post infection or display cytotoxic activity until 48 h.^41^ By this time, the sporozoites have undergone massive replication and maturation in the murine liver and will soon progress to the blood stage. If equivalent numbers of naïve CSP-specific CD8^+^ T cells or activated/memory transgenic T cells are transferred into mice that are later infected with *P. yoelii* sporozoites, only mice that receive activated/memory cells display a reduction in parasite rRNA.^41^ These results clearly show a requirement for activated/memory T cells for protective immunity against malaria. Given the rapid induction of effector functions by activated/memory CD8^+^ T cells (that is, IFN-γ^+^ after 4 h),^37^ it is likely that reactivation of these cells occurs within the liver. In human infection, merozoites are not released into the bloodstream until ~1 week postinfection so, perhaps, naïve parasite-specific T cells could contribute to the anti-parasite immune response in humans as they will have sufficient time to be primed, expand and acquire effector functions.

Once activated, naïve CD8^+^ T cells can differentiate into short-lived effector cells (SLEC) or memory precursor effector cells (MPEC).^54, 55^ The decision to turn into a SLEC or MPEC depends on the transcription factors the cell is expressing and the local environment.^54, 56^ Cells expressing T-bet and Blimp-1, or cells in the presence of high levels of inflammation (IFN-γ or IL-12) will typically develop into SLEC,^57^ while MPEC will develop under low inflammatory conditions and these cells will express transcription factors including Bcl-6, Id3 and Eomes.^54, 58, 59, 60^ In addition to differing transcription factor expression, SLEC and MPEC can be distinguished based on the expression of cell surface proteins KLRG1 and CD127.^61^ SLEC express high levels of KLRG1 but lack CD127 and as their name suggests these cells do not persist for long periods. Rather, these cells are very good effectors producing large amounts of IFN-γ, perforin and granzyme. MPEC are also capable of producing effector proteins (IFN-γ, perforin, granzyme) but to a lesser extent than SLEC, and these cells persist for long periods. Parasite-specific SLEC have been observed in the spleen following *P. yoelii* infection and while the number of these cells increases dramatically after infection, it declines after clearance.^62^ In contrast, MPEC can be observed throughout the infection and over time these cells develop into true memory T cells.^62^

Memory T cells provide long-term protection against their cognate antigen. Upon antigen re-encounter, these cells rapidly gain effector functions including cytokine production and lytic activity. Memory T cells have traditionally been divided into 2 subsets based on the expression of CD62L and CCR7: central memory T cells (T_CM_) predominantly reside in lymphoid tissues and express high levels of CD62L and CCR7 whereas effector memory T cells (T_EM_) express low levels of these markers and can be found in the spleen and peripheral tissues.^63^ Over the past decade, a third subset of memory T cells has been identified termed tissue-resident memory T cells (T_RM_). T_RM_ have been found in a variety of tissues including gut, skin, female reproductive tract, brain and kidney, and can be characterised by their inability to recirculate as well as high expression of and low KLRG1.^64, 65, 66^ CD103 expression has been observed on many of these cells but its expression is not absolute.^67, 68^

### Central memory T cells

Little evidence supports a major role for central memory T cells in protective immunity against malaria parasites. Following treatment with RAS, parasite-specific T_EM_ and T_CM_ can be found in the liver, with the majority of cells (96%) exhibiting a T_EM_ phenotype.^69^ Functional analyses reveal T_CM_ can produce IFN-γ after *in vitro* stimulation but not to the same extent as T_EM_.^69^ The ratio of T_CM_ to T_EM_ appears to have a significant role in protection against malaria. BALB/c mice are highly resistant to malaria, and following two treatments with RAS generate high numbers of CSP-specific T_EM_.^70^ In contrast, C57BL/6 (B6) mice, which are more susceptible to malaria produce a higher proportion of CSP-specific T_CM_.^70^ Similarly, vaccination studies using adenoviral vectors and modified vaccinia ANKARA (MVA) found mice receiving MVA expressing the multiple epitope string and thrombospondin-related adhesion protein (ME-TRAP) generated a large proportion of T_CM_ but these mice were not protected from malaria challenge.^71^ In contrast, mice vaccinated with adenovirus (AdV) vectors expressing ME-TRAP developed more T_EM_, and although these mice did not exhibit sterile immunity, they displayed some level of protection after challenge.^71^ In addition to highlighting the limited role for T_CM_ in protection against malaria, these data also show that the choice of vaccine vector can have a profound effect on the responding CD8^+^ T-cell population and in turn the effectiveness of the vaccine.

### Effector memory T cells

Effector memory T cells are ideally situated to respond to a malaria challenge due to their recirculation through the liver and rapid induction of effector functions. Large numbers of parasite-specific CD8^+^ T cells are required for protection against malaria possibly to ensure the small number of infected hepatocytes are found and eliminated.^5^ BALB/c mice immunised with DC, coated with a peptide from *P. berghei* CSP and boosted with *Listeria monocytogenes* expressing the same peptide developed large numbers of memory CD8^+^ T cells in the liver and spleen.^5^ Long-term protection was only observed in mice when their parasite-specific T cells made up more than 1% of the total peripheral blood CD8 T-cell population.^5^ Further delineation of CD8^+^ T-cell subsets revealed protection was associated with cells of a CD44^high^, CD45RB^low^, CD62L^low^ and CD122^low^ T_EM_ phenotype.^70, 72^ Intravital microscopy studies of these mice using cell tracker dyes or by injection of CD8α antibodies found the memory CD8^+^ T cells in the liver had a higher velocity compared with naïve CD8^+^ T cells and were amoeboid in shape. If these cells were purified from the immunised mice and transferred into mice infected with *Plasmodium* 2 days earlier, the transferred cells lost motility potentially indicating their capacity to recognise *Plasmodium* antigens in the liver.^73^

### Tissue-resident memory cells

Tissue-resident memory T cells are a non-circulating population of memory T cells that reside in non-lymphoid tissues and act as guards against pathogens.^64^ Parabiosis experiments, where the circulatory systems of two mice are joined, show a proportion of memory cells do not recirculate and hence are resident.^74, 75^ T_RM_ display a unique transcriptional^76^ and functional phenotype and may be distinguished from other memory T-cell subsets by their expression of CD69, a marker previously associated with recently activated T cells.^64^ High expression of CD69 appears to be required for the retention of T_RM_ in their target tissue as signalling via this molecule is required for the downregulation of sphingosine 1 phosphate (S1P) receptor.^77^ The S1P gradients usually draw T cells out of tissues but high expression of CD69 and hence low expression of S1PR1 prevents egress.^77^ Low expression of CCR7 on T_RM_ is also required for retention in tissues^76^ as its ligands CCL19 and CCL21 will draw T cells to the LN and spleen.

T_RM_ found in all tissues express a unique transcriptional profile characterised by high expression of CD244, cadherin 1 and 2, XCL1 and CTLA-4, and lack expression of Eomes.^78^ Until recently, a unique transcription factor had not been identified for T_RM_ but T_RM_ have now been shown to express the Blimp1 homologue Hobit (Homologue of Blimp1 in T cells).^79^ The combined loss of Blimp1 and Hobit prevents the formation of T_RM_ in various tissues including the liver as these transcription factors are required for the downregulation of S1PR1, CCR7 and KLF2, and tissue retention.^79^

T_RM_ patrol their target tissue for cognate antigen, and in the skin, for example, they extend dendrites in many directions.^80^ Skin-associated T_RM_ are relatively slow-moving compared with T cells in other sites but have the ability to produce IFN-γ and granzyme B, so they are poised to respond to an immediate threat or draw in cells from the circulation to provide added protection.^81, 82^ T_RM_ have recently been observed in the liver^66, 79, 83^ where they behave differently from those in the skin. These cells are found in the blood patrolling the sinusoids and are fast moving (10 μm min^−1^).^83^ Our studies reveal that these liver T_RM_ are essential for controlling sporozoite infection in RAS-immunised mice.^83^

Due to the recent identification of this cell subset, much of the data examining memory CD8^+^ T-cell responses in malaria infection will have to be reinterpreted with this population in mind. Many studies simply distinguished between CD44^+^ T_CM_ and T_EM_ based on CD62L expression, but T_RM_, like T_EM_ are CD62L^low^, so we suggest memory T cell subsets should be discriminated by additionally examining CD69 expression.^78^ T_CM_ would be the only subset expressing CD62L and T_EM_ could be distinguished from T_RM_ by their lack of expression of CD69. In this scheme, T_RM_ are at minimum CD44^+^, CD62L^−^ and CD69^+^ cells, and could also be distinguished from effector T cells by their lack of expression of KLRG1.

T_RM_ in most tissues are located separate from the vasculature^84^ so given the highly vascularised nature of the liver and the large blood volume passing through this organ, it was unclear if T_RM_ cells could differentiate and remain in the liver. Early studies using the adoptive transfer of transgenic T cells into recipient mice vaccinated with RAS identified novel gene transcripts expressed by memory T cells in the liver^85^ and retention of these memory T cells in the liver depended on the chemokine receptor CXCR6.^48^ Direct evidence that resident cells could form in the liver, however, came from an LCMV model. The LCMV immune chimeras were conjoined to naive mice via parabiosis surgery and 1 month later ~50% of the LCMV-specific CD8^+^ T cells in the liver parenchyma were found to be resident as they remained in their original host, failing to recirculate to the parabiont partner.^66^

Using RAS vaccination, we have recently demonstrated a requirement for T_RM_ in protection from malaria challenge.^83^ CXCR6^+^CXCR3^+^ T_RM_ (CD69^+^ CD62L^low^) that remained within the liver were generated in large numbers following vaccination and could be observed patrolling the sinusoids, as revealed by two-photon microscopy. Depletion of this subset using anti-CXCR3 antibodies abrogated sterile protection, providing evidence that RAS vaccination generates T_RM_ and that these cells are essential for protection from sporozoite challenge.^83^

## Mechanism of protection by CD8^+^ T cells

### Protection requires MHC Class I expression on hepatocytes

Given the plethora of data indicating a vital role for CD8^+^ T cells in malaria liver-stage immunity, it is still unclear how these cells mediate protection and eliminate parasites and/or infected hepatocytes. Bone marrow chimera studies show the elimination of parasites requires close contact between the responding CSP-specific CD8^+^ T cells and infected hepatocytes, as liver CD8^+^ T cells could not eliminate infected hepatocytes expressing mismatched MHC.^37^ CD8^+^ T cells possess several potential effector mechanisms that might control parasites, including production of cytokines such as IFN-γ and tumour necrosis factor (TNF)-α, or through direct lysis of infected hepatocytes by perforin and granzymes.^86^

### Role of cytokines, cytotoxic proteins and death receptors

IFN-γ has been shown to inhibit the liver-stage of disease^50, 51^ as injecting IFN-γ into mice can protect from challenge.^87^ Furthermore immunising IFN-γ receptor knockout mice with RAS does not result in protection.^88^ Studies analysing the response of endogenous parasite-specific CD8^+^ T cells in wild type and IFN-γ^−/−^ mice find expansion of CD8^+^ T cells is similar after *Plasmodium* infection but contraction of the T cells is attenuated in the knockout mice.^89^ Moreover, the recruitment of CD8^+^ T cells to the lung, liver and brain is distorted despite similar parasitemia.^89^ Some insight into the role of IFN-γ produced by CD8^+^ T cells came through studies where BALB/c mice received wild type or IFN-γ^−/−^ CD8 T cells specific for CSP.^90^ The mice were immunised with vaccinia virus expressing CSP and later challenged with sporozoites. Both groups of mice displayed a similar reduction in parasite load suggesting that IFN-γ is not required for parasite control. It should be noted, however, that large numbers of T cells were used for this study, which may not be biologically relevant, so additional studies will need to be performed using T-cell numbers that are more physiological.^90^ If the effects observed in mice where IFN-γ was neutralised^50^ were not due to CD8^+^ T cells then it is unclear which cell type is responsible for its production, but its effects may be exerted by increasing the expression of MHC class I on infected cells, enhancing recognition as targets.^91^ It may also synergise with TNF-α to activate local macrophages (reviewed in ref. 92).

Elegant studies conducted by the Harty lab reveal that the role of various cytokines and cytotoxic proteins in protection is heavily influenced by the recipient mouse strain and the *Plasmodium* species used.^70, 72^ B6 and BALB/c mice show striking differences in their response to RAS vaccination using *P. berghei* and *P. yoelii* sporozoites: BALB/c mice are 100% protected from high-dose challenge following vaccination with a single dose of *P. berghei* RAS.^70^ In contrast, the same vaccination strategy results in no protection in B6 mice, which need a booster dose to acquire protection.^70^ In both mouse strains, memory CD8^+^ T cells exclusively mediate protection. Protection against *P. yoelii* after immunisation with RAS is significantly lower in BALB/c and B6 mice, which display 10 and 0% protection respectively. Protection can be enhanced to 100% in BALB/c mice by performing a second vaccination with *P. yoelii* RAS but this only increases to 40% in B6 mice.^70^ The mechanism of protection by memory CD8^+^ T cells against *P. yoelii* and *P. berghei* was studied in prime-boost-vaccinated BALB/c mice deficient in IFN-γ, Perforin, TRAIL or FasL, or in which TNF was neutralised.^72^ Neither perforin, TRAIL nor FasL were required for protection against *P. berghei* ANKA infection but a lack of IFN-γ resulted in a 50% reduction in protection compared with wild-type mice; and TNF-α blockage diminished protection by 40%.^72^ IFN-γ and TNF-α also contributed to protection against challenge with *P. yoelii* sporozoites, as their absence resulted in a 35 and 85% decrease in protection, respectively. However, perforin was also involved in protection against *P. yoelii* parasites, as its deficiency diminished protection by 50%.^72^ These results indicate that CD8^+^ T cells utilise multiple effector mechanisms for the elimination of liver-stage malaria parasites, and that the relative contribution of these mechanisms varies according to the infecting parasite species and the host genetic background. This suggests that multifunctional T cells able to mediate all three contributing effector mechanisms (IFN-γ, TNF-α and cytotoxicity) may be the ideal goal of a vaccination.

### Lack of bystander elimination of parasites

The introduction of intravital imaging has provided unique insight into the behaviour of T cells in the liver following vaccination and/or malaria challenge. Antigen-specific and non-specific cells cluster around infected hepatocytes, suggesting that recognition of parasite antigens by antigen-specific CD8^+^ T cells can lead to the development of an inflammatory microenvironment and recruitment of other T cells to the site of infection.^93^ The potential for bystander killing of parasites was examined by injecting mice with two *P. berghei* strains, only one of which would be recognised by adoptively transferred transgenic T cells.^94^ CD8^+^ T cells did not kill the non-cognate parasite suggesting bystander effects do not have a major role in parasite control.^94^ It is unclear if CD8^+^ T cells can kill infected hepatocytes while still located within the sinusoids, as seen in hepatitis B virus (HBV) infection,^15^ or if they must extravasate across the endothelial barrier. CD8^+^ T cells can interact with antigen expressing hepatocytes by extending cytoplasmic projections through fenestrations in the endothelial layer to form trans-endothelial hepatocyte-lymphocyte interactions.^7^ These trans-endothelial hepatocyte–lymphocyte interactions are likely sufficient to trigger the release of IFN-γ and TNF-α but it is uncertain as to whether a complete immunological synapse forms allowing the controlled and directed release of perforins and granzymes to kill infected hepatocytes. Perhaps for CTL-mediated killing, extravasation of the T cells is required to allow a more stable interaction with the infected cell. Intravital imaging using fluorescently tagged transgenic T cells and sporozoites will provide much needed answers into the mechanism of parasite elimination in the liver.

## Towards an efficacious pre-erythrocytic malaria vaccine

Irradiated attenuated sporozoites represent one of the most effective vaccination approaches for protecting rodents and humans against *Plasmodium* infection.^95, 96^ This approach relies heavily on CD8^+^ T cells^50, 51^ (particularly T_RM_^83^), which kill parasites within the liver, but also utilizes antibody,^50^ which likely affects sporozoites during their migration from the skin to the liver. There are a number of logistical difficulties in developing an attenuated sporozoite vaccine, including the need for sterile sporozoites and a requirement for intravenous delivery,^96, 97^ and while these impediments have not precluded continued development of a clinically relevant sporozoite vaccine (see below), they have led to alternative streams of subunit vaccine development that may be more easily adapted to clinical use. These subunit approaches may utilize either cellular and humoral immunity or a combination of both to mediate their protection.

The first licensed malaria vaccine RTS,S is composed of HBV surface antigen virus-like particles genetically fused to truncated *P. falciparum* CSP. The CSP is a major surface protein expressed on sporozoites and contains CD4^+^ and CD8^+^ T-cell epitopes^98, 99^ as well as B-cell epitopes recognised by neutralising antibodies.^100^ Efficacy of the RTS.S vaccine has been largely correlated with anti-CSP antibody, suggesting this is its major effector mechanism.^101^ The RTS,S Phase III vaccine trial took place in seven African countries from May 2009. Over 15 000 infants and young children were enrolled and received either three doses of RTS,S 1 month apart, followed by a booster dose 18 months later, three doses of the vaccine followed by a booster control vaccine, or only control vaccines.^102^ Two age categories were established consisting of infants aged 6–12 weeks or young children aged 5–17 months. The efficacy of the vaccine against clinical malaria was lower in infants compared with young children, but was enhanced by the administration of a booster dose in both age categories.^103^ Despite boosting, the efficacy waned over time but regardless of this loss in efficacy the number of cases of clinical malaria was initially reduced.^103^ A smaller follow-up study of children enrolled in the phase II clinical trial was conducted 7 years after vaccination.^104^ Vaccine efficacy continued to wane such that after 7 years it was only 4.4%. In fact, in high exposure regions, malaria episodes were higher in the RTS,S group than the control group in year 5.^104^ This malaria rebound effect, where infection is merely delayed, may be due to the RTS,S protecting children against sporozites but not blood stage. Although initially protective, the study participants would develop natural immunity slower than those receiving the control vaccine and hence would be more likely to develop parasitemia over time. Further studies will need to be performed as this follow-up was conducted on a small group of individuals who received only 3 doses of the vaccine.^104^

Recombinant Adenoviruses (AdVs) have also shown promise as potential malaria vaccines. Not only are these vectors relatively simple to manufacture, they are cost-effective and can be easily stored.^105^ Moreover, AdVs have been shown in multiple settings to induce robust CD8^+^ T-cell and antibody responses in part due to their ability to trigger the innate immune system.^106, 107, 108^ Whether they induce liver T_RM_, however, has not been addressed. Mice vaccinated with rAdV5 expressing CSP from *P. falciparum* developed similar numbers of IFN-γ^+^ splenocytes compared with mice vaccinated with RTS,S, although the source of this cytokine was not determined.^109^ Similarly, antibody levels were equivalent between the two groups.^109^ Pre-existing immunity to AdV5 is common in malaria endemic regions, so alternate AdV vectors including AdV35 are also being trialled. AdV35 pre-existing immunity is less likely, but in immune individuals AdV35 encoding *P. falciparum* CSP induced poor antibody responses and limited CD8^+^ T-cell responses.^110^ Like AdV5, these vectors induce IFN-γ and antibody responses equivalent to those triggered by RTS,S in mice suggesting that these vectors may be effective in individuals lacking pre-existing immunity.^109^ While adenovirus-based vaccines have shown promise in animal models, their efficacy in humans has been disappointing. A phase IIa trial using a chimpanzee adenovirus (ChAdV63) and modified Vaccinia Ankara (MVA) expressing ME-TRAP or CSP protected 3 of 14 volunteers^111^ in one study and produced sterile immunity in 13% of individuals in another.^112^ T-cell responses were higher in those receiving ChAdV63-ME-TRAP and MVA-ME-TRAP, and vaccinees were more likely to develop sterile immunity but both vaccination strategies were not very protective.^112^ Given the growing evidence that liver T_RM_ are important for protection against liver-stage parasites, it will be interesting to see whether adenoviral vectors or MVA favour their generation.

### Whole-parasite vaccines

Vaccination approaches using whole sporozoites have been far more successful than subunit vaccines such as RTS,S and have achieved 100% protection. This may in part be due to a wider range of antigens being expressed by the whole parasite as opposed to one protein expressed in a subunit vaccine, though effector mechanisms generated by whole sporozoites, particularly T_RM,_^48, 83^ may be critical. Sporozoites can be attenuated by irradiation, genetically modified or controlled by treatment with anti-blood-stage drugs, and these vaccination approaches target distinct phases of the parasite life cycle.

#### Irradiated sporozoites

Attenuation of parasites using radiation was first demonstrated in the 1960s as a viable option for malaria vaccination in mice.^95^ Both the animal and human data indicate a high possibility of developing an effective pre-erythrocytic vaccine as humans, mice and non-human primates treated with RAS all develop sterile immunity following sporozoite challenge.^95, 113, 114^ This protection wanes over time but can be restored by secondary or booster vaccinations.^115^ Using this approach, it is vital that the RAS remain viable enabling the parasites to infect hepatocytes as heat-killed sporozoites that lack the capacity to infect hepatocytes do not provide protection.^116^ Although this protective effect was first observed in the 1960s in mice^95^ and confirmed in humans a decade later,^113^ there is limited data concerning the mechanistic requirements for immune protection in man. The data from humans immunised intravenously with RAS suggest the magnitude of antibody responses targeting the parasite correlate with protection,^96^ but correlation is not evidence of a functional role for this effector mechanism. As described above, RAS immunization of mice leads to the expansion of CD8^+^ T cells that are largely responsible for protection. Similarly, immunization of non-human primates triggers the expansion of parasite-specific CD8^+^ T cells.^97^ Thus, it is highly likely that this population of T cells also contribute to RAS-mediated immunity in humans. An irradiated sporozoite vaccine known as PfSPZ, generated through the large-scale production of cryopreserved, aseptic irradiated sporozoites is currently being tested in clinical trials.^96, 97, 117^ These sporozoites are attenuated so that they cannot proceed past the late liver stage. Initial trials showed that this vaccine was well tolerated, but only 2 of 44 volunteers were protected against sporozoite challenge.^97^ This was thought to be due to the subcutaneous and intradermal vaccination routes used in the trial so additional studies were conducted in non-human primates using the intravenous route. Vaccination via this route induced long-lasting CD4^+^ and CD8^+^ sporozoite-specific T-cell responses in the liver prompting another human trial using PfSPZ, in this case delivered intravenously. All volunteers given five doses of PfSPZ were protected from challenge 3 weeks after the final vaccination as were 66% of the volunteers receiving four doses.^96^ The durability of this vaccine was tested in an additional study, which used a higher dose of PfSPZ.^117^ Vaccinees were challenged at 3 or 21 weeks after receiving four doses of PfSPZ and 7/9 and 3/4 individuals were protected respectively. Of those that remained parasite free, five were rechallenged at 59 weeks and all were protected, resulting in a cumulative vaccine efficacy of 55%.^117^ These results are encouraging but may be difficult to achieve in the field as human trials indicate at least 4 doses of 135 000 irradiated sporozoites are required for protection. Generating sufficient numbers of irradiated, cryopreserved sporozoites for a large proportion of the world's population living in endemic areas may not be feasible or indeed cost-effective, so alternate whole-parasite vaccines are being investigated.

#### Drug-attenuated sporozoites

The use of drug-attenuated parasites was first explored as a vaccination strategy in the 70s and 80s in rodent malaria models.^118, 119^ Using this approach, sporozoites can infect the liver, develop into merozoites but are controlled at the blood stage using anti-malarial drugs such as chloroquine. This allows the host immune system to be exposed to many antigens expressed at various stages of the parasite life cycle. Although this approach has been very successful in animal models, it has only recently been tested in humans. Ten volunteers were immunised three times with *P.falciparum* by receiving bites from infected mosquitoes while receiving chloroquine. All 10 volunteers developed sterile immunity when challenged 8 weeks after the final immunisation,^120^ and of those 4 of 6 were still protected when challenged a second time 2 years later.^121^ Analysis of immunity in the blood samples suggested protection from challenge was linked to the induction of memory CD4^+^ and CD8^+^ T cells that produced IFN-γ, TNF-α and IL-2 when exposed to infected RBC. A second study implicated a role for CD4^+^ T cells in the blood and their expression of the degranulation marker CD107a in protection as well as CD8^+^ T cells producing Granzyme B.^122^ Intrahepatic immune responses were not analysed in either of these studies.

Using drug-attenuated vaccines instead of RAS is advantageous as only a few mosquito bites are required for protection (12–15 bites per vaccination versus 1000 bites for RAS). Of the many anti-malarial drugs available, only chloroquine and mefloquine have been used in drug-attenuated malaria vaccination. Resistance to anti-malaria drugs is widespread in endemic regions thus it will be necessary to assess drug resistance in these areas before this approach can be employed.

#### Genetically attenuated sporozoites

Parasites can be attenuated through deletion of genes that are required for liver-stage development allowing priming of the immune system during the pre-erythrocytic stages without progression to the erythrocytic stage. The use of genetically attenuated parasites (GAP) as an effective vaccine was first demonstrated in 2005 when sporozoites that lack the UIS3 gene, which is required for early liver-stage development, completely protected mice from sporozoite challenge.^123^ These data suggest the use of GAPs that develop further in the liver stage, that is, late liver phase, may generate more protective immune responses.^124^ This is believed to be because late arresting parasites expose the immune system to a broader range of antigens than those arrested in the early part of the liver stage, allowing for a more diverse immune response capable of targeting both the pre-erythrocytic and erythrocytic stages.

To develop a safe and effective GAP, there must be complete attenuation in the liver. Candidate genes including UIS3, UIS4 and P52 have all been deleted in murine *Plasmodium* strains and vaccination with these GAPs has proven effective.^123, 125, 126^ Sporozoites lacking both P52 and its relative P36 were the first GAP generated in *P. falciparum* for use in human trials. Attenuation appeared complete when volunteers received five bites from infected mosquitoes carrying these GAPs but one volunteer became positive for blood-stage malaria after receiving 200 bites, indicating that the mutant was not completely attenuated.^127^ Continuing studies in mice have identified many more gene targets for the production of GAPs but only one target has shown promise for human trials due to its absolute attenuation in the liver. GAPs lacking the sap1 gene are completely attenuated in the liver even when given at extremely high doses as are triple knockout GAPs lacking sap1, p52 and p36.^128, 129^

The data generated in mouse models using GAPs have demonstrated a requirement for the induction of CD8^+^ T-cell responses to develop sterile immunity.^130, 131^ Given the data implicating T_RM_ in RAS-induced immunity,^83^ it seems likely these cells are also involved here. These parasite-specific CD8^+^ T cells appear to protect using IFN-γ because a higher frequency of IFN-γ-producing cells can be found in the livers of mice treated with GAPs compared with mice treated with RAS, but a direct correlation between IFN-γ production and sterile immunity has not been demonstrated.^130^ Similar data obtained from humans treated with GAPs indicate a potential role for IFN-γ produced by CD8^+^ and CD4^+^ T cells.^127^

## Concluding remarks

Mouse models have provided valuable insights into the requirements for inducing and maintaining sterile immunity against malaria. The need for large numbers of multifunctional (IFN-γ^+^, TNF-α^+^, Perforin^+^) memory CD8^+^ T cells and the potentially important role of liver-associated T cells is evident from multiple studies using different transgenic T-cell populations, recipient mouse strains, *Plasmodium* species and vaccination approaches. Moreover, these data are consistent with many malaria vaccine trials conducted in humans. Thus, it is essential that new malaria vaccines target the generation of these T cells to maximize efficacy. Although it is not feasible to test the role of specific T-cell populations in humans (via depletion for example) or to directly assess interactions in the liver, it is vital that research focuses on examining a link between the immune populations shown to be important in rodent immunity to malaria and those required for protection in humans.
